# Assessing LLIN distribution implementation using evidence-informed intervention core elements: a qualitative study in a resource-constrained setting

**DOI:** 10.1186/s12913-024-11223-5

**Published:** 2024-07-09

**Authors:** Phyllis Dako-Gyeke, Emmanuel Asampong, Franklin N. Glozah, Ruby Hornuvo, Philip Teg-Nefaah Tabong, David Gittelman, Adanna Nwameme, Benjamin Oteng, Nana Yaw Peprah, Gloria M. Chandi, Philip B. Adongo

**Affiliations:** 1 Department of Social and Behavioural Sciences, School of Public Health, University of Ghana, Accra, Ghana; 2 National Malaria Elimination Programme, Accra, Ghana; 3 Ghana Health Service, Ga North Municipal Health Directorate, Greater Accra, Ghana; 4 Advisor, Health Campaign Effectiveness Coalition, Task Force for Global Health, Decatur, GA USA

**Keywords:** Long lasting insecticide treated nets (LLIN), Evidence informed interventions, Core elements, Taxonomy, Ghana, Malaria, Implementation, Campaign

## Abstract

**Background:**

The National Malaria Elimination Programme implements the mass LLIN Distribution Campaigns in Ghana. Implementation science promotes the systematic study of social contexts, individual experiences, real-world environments, partnerships, and stakeholder consultations regarding the implementation of evidence-informed interventions. In this paper, we assess the core elements of the mass LLIN distribution campaign in a resource constrained setting to learn best implementation practices. Three core domains were assessed through the application of Galbraith’s taxonomy (i.e., implementation, content, and pedagogy) for evidence-informed intervention implementation.

**Methods:**

Six districts in two regions (Eastern and Volta) in Ghana participated in this study. Fourteen Focus Group Discussions (FGDs) were conducted across these communities. Eligible participants were purposively sampled considering age, occupation, gender, and care giving for children under 5 years and household head roles. All audio-recorded FGDs were transcribed verbatim, data was assessed and coded through deductive and inductive processes. NVivo software version 13 was used for the coding process. Themes were refined, legitimized, and the most compelling extracts selected to produce the results.

**Results:**

Sixty-nine (69) caregivers of children under 5 years and sixty (60) household heads participated in the FGDs. All caregivers were females (69), whilst household heads included more males (41). Core elements identified under implementation domain of the LLIN distribution campaign in Ghana include the registration and distribution processes, preceded by engagement with traditional authorities and continuous involvement of community health volunteers during implementation. For pedagogy domain, core elements include delivery of intervention through outreaches, illustrations, demonstrations, and the use of multiple communication channels. Core elements realized within the content domain include information on effective malaria prevention, and provision of information to enhance their self-efficacy. Yet, participants noted gaps (e.g., misuse) in the desired behavioural outcome of LLIN use and a heavy campaign focus on women.

**Conclusion and recommendations:**

Although the implementation of the mass LLIN distribution campaigns exhibit components of core elements of evidence informed interventions (implementation, content and pedagogy), it has not achieved its desired behavioural change intentions (i.e. continuous LLIN use). Future campaigns may consider use of continuous innovative pedagogical approaches at the community level and lessons learnt from this study to strengthen the implementation process of evidence-based health interventions. There is also the need for standardization of core elements to identify the number of core elements required within each domain to achieve efficacy.

**Ethical approval:**

Ethical clearance was obtained from the Ghana Health Service Ethics Review Committee (GHS-ERC: 002/06/21) before the commencement of all data collection.

**Supplementary Information:**

The online version contains supplementary material available at 10.1186/s12913-024-11223-5.

## Contributions to the literature

- This study highlights the importance of community engagement in the implementation of evidence-based health interventions in resource constrained settings.
- Health programme implementers recognizing that communities are experts in their own lived experience and possess cultural competence, thus engaging them in the development of solutions can enhance the relevance of interventions and facilitate uptake and sustainability.
- Findings in this study reiterate the need to make room for the uniqueness of contexts across time and space.

## Introduction

Long-lasting insecticidal nets (LLINs) continue to contribute towards a reduction in the global malaria burden [1]. LLIN is an evidence-informed prevention tool recommended widely for use among populations at risk of malaria [2, 3]. To maintain universal LLIN coverage, countries implement intermittent mass free net distribution campaigns, alongside continuous multiple channel distributions (e.g., antenatal clinics, EPI, etc.) [1]. The Mass LLIN distribution campaign in Ghana is implemented through a set of activities conducted (i.e. pre-registration of persons and sleeping places, door-to-door distribution of LLINs with ‘hang-up’ activities by volunteers and post-distribution ‘keep-up’ behaviour change communication activities at the community level) [4], that is among a group of people with some kind of shared social identity [5]. Similar to the implementation of other evidence-informed interventions, mass LLINs implementation is executed through interpersonal processes, which facilitate encounters between healthcare professionals and the target populations [6] to ensure the intervention produces its main effects (i.e. LLINs access and use to eliminate the burden of malaria) [7].

The National Malaria Elimination Programme (NMEP) is responsible for the implementation of LLIN Distribution Campaigns in Ghana [7]. The distribution of LLINs is the core intervention for malaria control in Ghana, as it provides protection against mosquito bites and reduces the risk of malaria both at individual and community levels. The LLINs are distributed nationwide mainly during the Point Mass Distribution (PMD) campaigns, and through continuous channels for pregnant women, children below 5 years of age and primary school children (i.e. Antenatal care (ANC), Child Welfare Clinics (CWC), and primary schools) [8].Between 2010 to 2012, there was a nationwide LLIN door-to-door Mass Distribution and the Hang-up Campaign. This was followed by another Mass Campaign in 2018, which employed the use of technology in the capture and management of registration and Distribution data via mobile application software (i.e. NetApp). In 2018, social behaviour change communication (SBCC) activities were planned to run throughout pre- campaign, campaign and post campaign activities.

Though there have been improvements in overall LLIN ownership over the years, the 2019 Ghana Malaria Indicator Survey shows that 67% of Ghanaian households have access to LLIN but only 43% of the Ghanaian household population slept under LLINs the night before the survey. This shows that, there is a gap between LLIN access and LLIN use in Ghana. In addition, the NMEP’s strategic goal (2021–2025) of 80% utilisation among pregnant women and children under five is yet to be met [9, 10]. Some identified barriers to LLIN use among community members include limited social and behaviour change communication (SBCC) activities, knowledge gap in relation to malaria prevention, inability to hang LLINs in many community households due to housing type and sleeping places and lack of continuous malaria education [11, 12]. Although there has been progress in overall LLIN ownership in Ghana, the implementation process has yet to lead to the needed behaviour change and improve the LLIN use among target groups [9].The science of implementation promotes the systematic study of social contexts, individual experiences, real-world environments, partnerships, and stakeholder consultations regarding the implementation of evidence-informed interventions [6]. This study involves gathering program practice information from the perspective of end-users, which is useful for assessing evidence informed intervention during its implementation within a real-world setting.

Evidence informed interventions exhibit identifiable core elements which guide the needed balance between adaptation and fidelity regarding the implementation. These core elements are features that embody the theory and internal logic of the evidence informed intervention and most likely lead to the intended intervention outcomes [13].They highlight features required to deliver the intervention, cultural considerations to be made during the intervention implementation and specify boundaries between programmatic activities that should not be altered and those that can be modified [14].

An extensive review by Galbraith and colleagues, categorizes these core elements into three domains: implementation, content, and pedagogy. Implementation domain includes items describing the intervention delivery processes, such as support from stakeholders. Content domain involves facets of behaviour change theory that can influence positive intentions for the performance of protective behaviours. The pedagogy domain consists of how interventions are delivered (e.g., outreach, modelling, and demonstrations). All domains are expected to enhance interpersonal relationships and communications within sociocultural contexts that shape the acceptability and meaningfulness of healthcare interventions [6, 10].

In this paper, we assess the core elements of the mass LLIN distribution campaign in Ghana to learn best implementation practices, by applying Galbraith’s taxonomy for evidence-informed intervention implementation. The application of Galbraith’s taxonomy to assess the core elements of the LLIN implementation process in Ghana is to determine if the LLIN implementation process in Ghana encompasses the required characteristics needed for the efficacy of evidence-based behavioral interventions. Though there have been improvements in overall LLIN ownership over the years, the 2019 Ghana Malaria Indicator Survey shows there is a gap between LLIN access and LLIN use in Ghana. This study was conducted as a Pre-Point Mass LLIN distribution activity for 2021, which used community members’ experiences of the previous implementation exercise. Learnings from this study can guide the efficacy of future implementation of mass LLIN distribution campaigns within other resource-constrained settings.

## Materials and methods

### Study design

The larger study employed a concurrent triangulation mixed methods research design, involving the use of participatory approaches within an implementation research framework. This paper however presents findings from the qualitative data collected at the baseline phase of the study. The qualitative component (In-depth interviews (IDI), Key informant interviews (KII) and Focus group discussions (FGD) of the study employed phenomenology to assess LLINs campaign implementation processes at the community level.

Qualitative research offers systematic ways of exploring the sociocultural context within which evidence-informed healthcare interventions are delivered, whilst providing means of gathering and analysing perspectives, features of interactions between community members and practitioners, language used in health information, and tools to understand end-users’ expectations. This approach puts the community members’ “lifeworld” at the centre (e.g., socio-economic, cultural, historical and geographical context) and gain understanding of their ability to take control, or have agency, through interactions with healthcare providers and policy-makers [6].

### Study setting

**Fig. 1:**
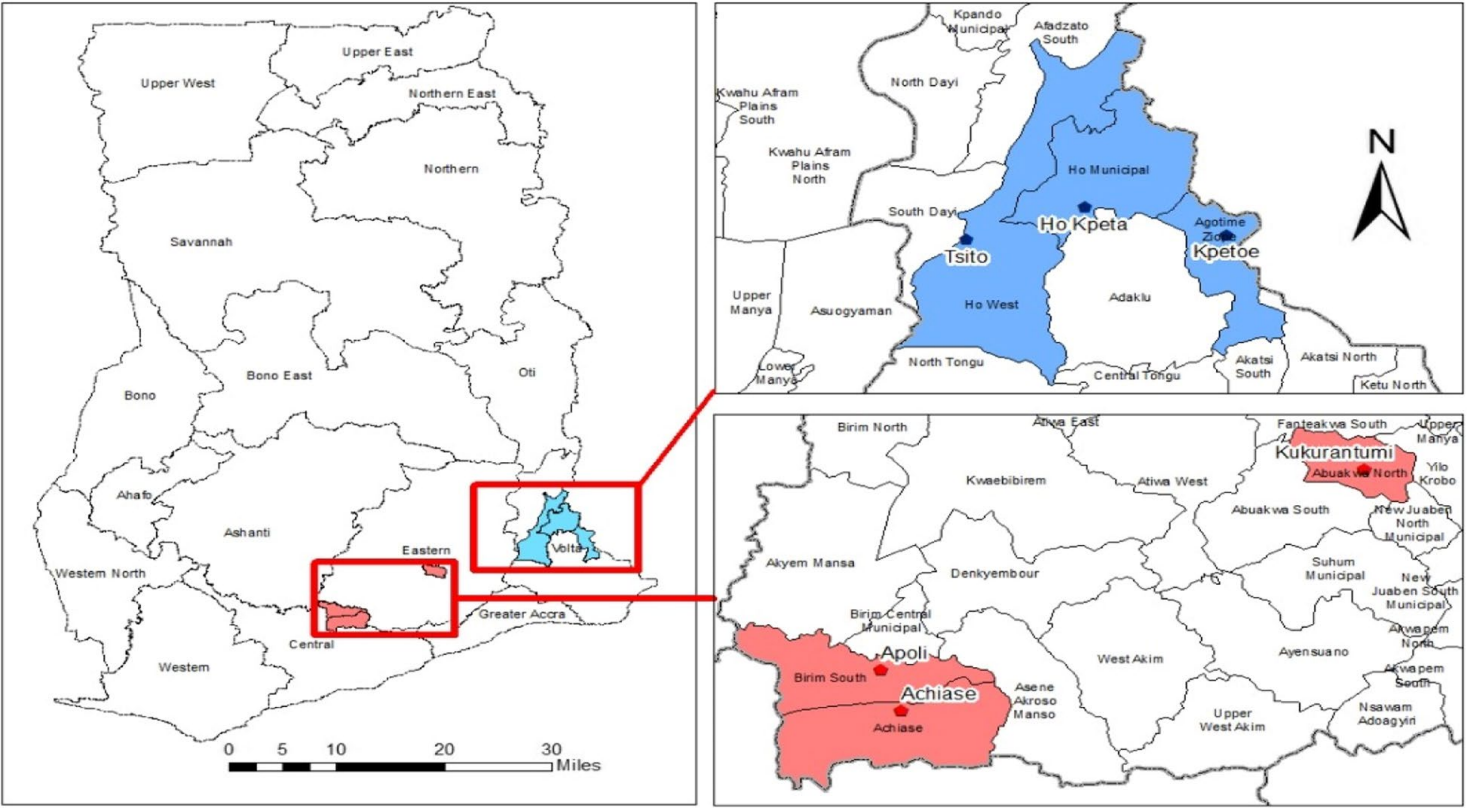
Location of the six study districts in the Eastern and Volta Regions of Ghana

A total of six districts (one community per district) across two regions (Eastern and Volta) in southern Ghana participated in this study. These were communities in districts as shown in Fig. 1, where the 2021 Point Mass Distribution (PMD) campaigns of LLINs were at different stages of pre-implementation. The selected districts are recorded to have the highest prevalence of malaria in the year 2021 (Ho West, Tsito-90%; Ho, Takla Hokpeta-75%; Agortime Ziope, Kpetoe-100% in the Volta Region; Birim South, Apoli-94%; Achiase, Achiase-94%; Abuakwa North, Kukurantumi-93% in the Eastern Region) (The highest prevalence of malaria as reported in the District Health Information Management System (DHIMS2).

### Study population

The study population comprised of adult men and women from these communities ages 18 years old and above, who have lived in the respective communities for more than 5 years and were present during the 2018 LLIN distribution campaign**.**

### Sample size and sampling technique

The sample size for the qualitative component (caregivers and household heads) conducted among community members was *n* = 129, who participated in fourteen FGDs across these communities. To achieve the study objectives, eligible participants were purposively sampled based on characteristics such as age, occupation, gender, caregivers of children under 5 years, and household heads. This sample size was achieved after saturation was reached [15].

### Data collection tools and techniques

Using an FGD Guide (Appendix 1), Fourteen (14) FGDs were organized to assess the sociocultural contexts within which the LLINs campaign was delivered. This provided opportunity to gather perspectives on interactions between community members and practitioners during the implementation of the 2018 mass LLIN distribution campaign. The FGD guide was developed and reviewed by the research team, and the tool was pre-tested with a sample of household heads and caregivers in the Greater Accra Region. The FGD guide was revised as needed based on feedback received prior to the data collection.

FGDs involved purposively selected household heads (6 FGDs) and caregivers of children under 5 (8 FGDs). Each FGD comprised 5 to 12 participants. An ethics approval letter to conduct the study was obtained from the Ghana Health Service Ethics Review Committee and submitted to the Volta and Eastern Regional Directors through in-person visits, and permission was granted. The district directors then led the project team to initiate community entry processes. Eligible participants were recruited, contacted and FGDs were conducted by six (4 males and 2 females) trained qualitative research assistants (A.O., C.E.G, K.A.C., S.T.C., A.A., and A.A.A.A.) within a relaxed and convenient atmosphere away from any interference. Each FGD lasted between 45 min to 1 h, 40 min. FGDs were conducted in English, Ewe and Akan depending of the primary language of the participants. All COVID-19 protocols were observed, including social-distancing, wearing of facemasks, and use of alcohol-based hand sanitizer. All research assistants have a master’s degree in the field of health, applied sciences and social sciences and have more than 2 years of field experience in conducting qualitative research.

### Qualitative data management, processing and analysis

All audio recorded FGDs were transcribed verbatim and augmented with researchers’ field notes written through observation and during the FGDs. Audio recordings in local dialects were translated and transcribed in English language and was reviewed by two independent consultants. The data resulting from transcriptions were assessed and coded through both deductive and inductive processes [16]. A codebook was first developed for all the FGDs. These were based on the research objectives, and FGD guides. The codebook development involved three qualitative experts who independently reviewed the various components of the codes to ensure they aligned with the datasets. The codebook included code number, the code, code names, the definition of codes and examples of use. All codes initially developed were revised throughout the coding process to include emerging ones. The involvement of three qualitative experts who independently reviewed and validated each stage of the qualitative analysis helped reduce any potential biases.

To ensure effective data analysis process and intercoder reliability, four (4) qualitative research assistants were selected and trained on the study objectives, the qualitative component, as well as the collaborative coding process using the NVivo software version 13 (QSR International). The four research assistants assisted with the coding process however, the analysis for this paper was primarily conducted by PDG. All codes were entered into the NVivo software version 13 for coding of the data. Also, a collaborative platform was created to support the research assistants in addressing emerging issues and compare emerging themes and findings. The coded datasets were then reviewed to align with the complete dataset, merged and analyzed to develop themes and sub-themes.

The themes were then refined and independently validated, where the most compelling extracts were selected to produce the qualitative result section for this study. To ensure accuracy, credibility and validity of the qualitative data, the findings were presented to study participants (member checking) in the various study communities, and there was no request from community members for changes to be made to the finding.

### Ethical considerations

Ethical clearance was obtained from the Ghana Health Service Ethics Review Committee (GHS-ERC: 002/06/21) before the commencement of all data collection. All qualitative research assistants received specific training before data collection per the study’s training protocol. Further, all the methods were performed in accordance with relevant guidelines and regulations.

### Informed consent

All study participants provided written informed consent in advance. The information and consent documents were written in simple English; however, for better comprehension, research assistants were present during the informed consent process to explain in local dialects (Ewe and Akan) and answer any questions that the participants might have. Those who consented to participate in this study signed (or placed a thumbprint on) an informed consent form. All participants were assured that the information provided will be handled confidentially and research findings will be reported with complete anonymity.

## Results

### Description of community members involved in this study

As shown in Table 1, a total of sixty-nine (69) caregivers of children under 5 years (CG) and sixty (60) household heads (HH) were sampled in the six communities across Eastern and Volta Regions of Ghana to participate in focus group discussions. All caregivers who participated were females (69), whilst household heads included more males (41). Also, many (27) of the caregivers were aged between 30 to 39 years, with 32 household heads aged more than 50 years. The highest level of education recorded among caregivers was secondary level (46). A few (8) had attained tertiary level education. The majority (91) of the participants (caregivers and household heads) were married and the highest number of children recorded per participant was 8 for caregivers, and 9 among household heads. Both household heads and caregivers were mainly traders/artisans (49) and farmers (44), most of whom (95) had stayed in their respective communities for more than 10 years.

**Table 1:** Socio-Demographic Characteristics of Participants (Caregivers of Children Under 5years and Household Heads)

**Number of Participants**			
**Characteristics of Participants**	**CG (FGDs)**	**HH (FGDs)**	**Total (CG &HH )**
**Community of Residence**			
***Eastern Region ***			
Kukurantumi	-	20	20
Achiase	16	-	16
Apoli	18	16	34
***Volta Region***			
HoKpeta-Takla	22	13	35
Tsito	-	11	11
Kpetoe	13	-	13
**Total**	**69**	**60**	**129**
**Sex**			
Female	69	19	88
Male	-	41	41
**Total**	**69**	**60**	**129**
**Age**			
>20years	7	-	7
20-29years	25	-	25
30-39years	27	13	40
40-49years	6	15	21
50+years	4	32	36
**Total**	**69**	**60**	** 129**
**Educational Level**			
No formal education	-	9	9
Primary	15	12	27
JHS/Secondary/Middle School	46	31	77
Tertiary	8	8	16
**Total**	**69**	**60**	**129**
**Marital Status**			
Single	14	4	18
Cohabiting	10	-	10
Married	40	51	91
Divorced/Widowed/Separated	5	5	10
**Total**	**69**	**60**	**129**
**No. of Children**			
<5	56	1	57
5-9	13	39	52
10+	-	20	20
**Total**	**69**	**60 **	**129**
**Occupation **			
Unemployed	17	2	19
Petty trading/Artisan	25	24	49
Farming	19	25	44
Formal Employment	7	5	12
Retired	1	4	5
**Total**	**69**	**60**	**129**
**Length of Stay in Community**			
<5years	3	-	3
5-9years	21	10	31
10+years	45	50	95
**Total**	**69**	**60**	**129**

This study presents findings categorized into three domains of evidence-informed interventions (implementation, content, and pedagogy) using Galbraith’s taxonomy.

### Implementation Domain

Under the implementation domain of Galbraith's taxonomy, we use community members’ experiences to highlight the LLIN distribution stages and community engagement processes. Themes identified under the implementation of the LLIN distribution campaign that aligns with Galbraith's taxonomy are presented in summary thematic table (Table 2).

**Table 2:** Thematic table (Core elements identified within the LLIN distribution campaign in Ghana which aligns with components of Galbraith’s Taxonomy)

**Domain**	**Themes**	**Galbraith's implementation categories of core elements that aligns with identified themes**
Implementation domain	Household Registration and LLIN Distribution	Have clearly defined target population for whom intervention is appropriate
Implementation domain		Group size
Implementation domain		Intervention location appropriate for target population
Implementation domain		Intervention dosage (amount), number of sessions
Implementation domain	Community Support for LLIN Campaign Implementation	Ensure necessary support from stakeholders/gatekeepers
Implementation domain		Select providers, volunteers and key staff with desired characteristics e.g. passionate about serving the client, respect for clients, peer, ethnically matched, can build rapport
Content domain	Information on effective prevention of malaria in the community	Influence social norms for protective behavior
Content domain		Intervention content that is appropriate for target population (e.g. culturally, developmentally, gender appropriate)
		Influence expectancies (e.g. consequences or benefits) for protective behavior
Content domain	Self-efficacy for Appropriate LLIN Use	Influence self-efficacy of protective behavior
Content domain		Influence cognitions for positive behaviors (not otherwise specified e.g. attitudes)
Content domain		Provide skills training for correct use of risk-reduction supplies or techniques
Pedagogy domain	Outreach, illustrations, and demonstrations	Delivered using multiple modalities/delivery strategies/levels
Pedagogy domain		Demonstration/modeling
Pedagogy domain		Outreach
		Distribution of information (brochures, posters)
Pedagogy domain	Communicating through Multiple Channels	Social event
Pedagogy domain		Use of electronic technology (e.g. video)
Pedagogy domain		Social marketing/mass media, e.g. TV, radio, billboards
Pedagogy domain		Empowerment/target audience has ownership of intervention
Pedagogy domain	Strengthening Campaign Through Continuous Engagement	

#### Household registration and LLIN distribution

Although not all community members could appropriately mention the intervals between previously implemented LLIN distribution campaigns, they described the various stages of the LLIN campaign to include registration at the household level (i.e., counting the number of people who live within particular households, marking the various housing structures, and collection of needed demographic information). Below are some quotes shared by participants:

*“They visited every house and counted us; the number of people who eat together” (R10, Caregivers FGD, Kpetoe).*

*“When they visit the houses, they write names of people; the number of people in the household is the criteria for sharing the LLINs” (R9, Caregivers FGD, Kpetoe)*

*“Before the nets were distributed, they came house to house to write the names so when the nets arrived an announcement was made and we all met at Adwenease” (R2, Caregivers FGD, Apoli-Ningo)*

Participants mentioned that the registration process was then followed by distribution, in which community members received coupons to redeem the number of bed nets each family was eligible for. For example:

*“...they brought a machine. They will mention your name and ask you to place your hand on the machine. If your name is on the machine, then they will remove it [LLINs] for you and you leave” (R13, Caregivers FGD* *,* *Kpetoe).*

*“They wrote down our names and later called me for it. I took mine in the church” (R8, Household Heads FGD, Kukurantumi)*

*“They came and wrote down our names and later brought them to us” (R10, Household Heads FGD, Kukuruantumi)*

#### Community support for LLIN campaign implementation

Community members across the selected districts mentioned how the campaign implementers involved the communities, either through seeking permission, or by highlighting various roles played by diverse community stakeholders. First, the Ghana Health Service personnel/NMEP solicited support from traditional leaders, especially the chiefs. This was mainly done as a community entry gesture, preceding the LLIN distribution campaign team. For instance, participants stated that the [health workers]/NMCP paid courtesy calls at the chief’s palace to first seek their permission. As community members described:

“*So they [GHS] …visit the community to ask permission from the chief”. Our chief also does not decline the agenda. He grants the permission and they enter the communities and visit us…Before they come, they will inform the chief before they enter the houses and roam. There are times that they will beat the gong that the nurses will come and interact with the children*” *(R7*, *Caregivers FGD, Kpetoe)*.

*They announce and inform the chief and the Unit Committee Members”* *(R5,* *Caregivers FGD, Kpetoe* *).*

Whilst the traditional leaders were contacted for purposes of permission into the community, health volunteers were recruited to actively support the registration and distribution processes. Community members stated:* “Before the distribution, they (campaign implementers) select the people they will work with (R4, Caregivers FGD, Apoli-Ningo)*

Below are other comments from community members:

*“One nurse brought the nets and gave them to a lady in our community and she shared it for us. That is what I saw…I know someone who helped with the distribution ... Before the campaign they came to her earlier, and she said she would not do anything on those days so she can help them go around to share the nets” (R4, Caregivers FGD, Achiase)*

*“I know someone who helped. When they started the registration, she went around to do the household registration and asking the number of persons in a house” (R1, Caregivers FGD, Achiase).*

Other caregivers mentioned the benefits of using the community volunteers during the Point Mass Distribution LLIN campaigns. They believed that volunteers had local knowledge and are familiar with the geographic terrain of the various communities:

*“During the net distribution here, the community members know the town more than the nurses so we have volunteers who help with the programs in the community. So the volunteers join the nurse since they know the town every well and all the corners. Together they go to the houses to write the names of the household heads and the number of persons”(R7, Caregivers FGD, Apoli-Ningo)*

*“The nurses are visitors and they do not know some places. The nurses are also not many, so the community has delegated some members to help because some towns are hidden in the forest. People from there come to the clinic here but the nurses do not know where they are coming from. Without the town people they will not be able to do the work.*” *(R1*, *Caregivers FGD, Apoli Ningo)*

However, some participants expressed concerns about the disrespectful attitude of some community members towards the health volunteers. Some household heads also argued that community should be involved in volunteer selection, whilst others called for training to be done for the volunteers before the execution of the campaign:

*“...They do not respect the volunteers...This happens a lot when volunteers ask them questions, they are upset but when the nurses ask the question, they are polite to them because they know they are government workers they give them respect but community members do not respect each other” (R1, Caregivers FGD, Apoli Ningo*)

*“Before the distribution we need to train the volunteers to teach them on how the nets will be shared so that when they go to the homes, they know what to do...”(R7, Household Heads FGD, Kukurantumi)*

*“I think if they recruit people from the village, you can inform us to help you recruit local people maybe 3 or 4 people in the community which will help a lot” (R2, Household Heads FGD, Tsito).*

### Content domain

The content domain involves facets of behaviour change theory included as components of the intervention delivery. Such content is expected to provide culturally-appropriate health information for the target population and address feasible social, ecologic or structural influences, offer skills to perform protective behaviours, and enhance communication. For this domain, we report information that was provided regarding how the community can effectively prevent malaria, as well as self-efficacy for appropriate LLIN use. Regardless, the conversations also clearly indicated inadequate behaviour change regarding the LLIN use within the various communities.

#### Information on effective prevention of malaria in the community

Participants recalled information that was provided about how they could effectively prevent malaria through LLIN use, whilst ensuring their surroundings are clean to prevent breeding of mosquitoes. Below are statements from community members highlighting the health information provided:

*“They also spoke about malaria and how to effectively use our nets to prevent mosquito bites”(R4, Caregivers FGD, Hokpeta- Gbogame)*

*“They tell us to sleep together with our kids under a treated mosquito nets to avoid getting malaria (R6, Caregivers FGD, Hokpeta- Gbogame)*

*“We should keep our homes and surroundings clean to avoid breeding of mosquitoes”(R11, Caregivers FGD, Hokpeta-Gbogame)*

*“They spoke about malaria, they said we should keep our surroundings clean and get rid of all waters in cans to avoid breeding of mosquitoes and sleep in treated nets with our families to prevent getting infected with malaria”(R3, Caregivers FGD, Hokpeta-Takla).*

*“Sometimes they come house to house to educate us on how to prevent malaria.. If you have empty tins of milk or tin tomatoes you have to tie them in rubber and dispose them at the refuse dump but if you leave them around and it rains in them they breed mosquitoes and the bite you. Also they tell us to sleep under the net, and teach us how to enter the nets at night and how to tuck them in the bed so mosquitoes do not enter” (R1, Caregivers FGD, Apoli-Ningo)*

Although this information was given, participants quickly noted that, in some instances there was not enough enabling environment to support the application of the knowledge provided through the campaign:

*“There are some places without waste bins or places to dump rubbish. We all go to Methodist to dump our rubbish. Some people just dispose the rubbish any how because the feel they place is too far for them to go. We are pleading on our leaders to bring waste management tracks here so that it will help reduce the dirty in the environment. This place is very large yet we have only one place of disposing wast*e” *(R7, Caregivers FGD, Achiase)*

#### Self-efficacy for appropriate LLIN use

Community members could recall campaign messages aimed at strengthening their efficacy to appropriately use LLINs in the community. For instance, participants described how the LLINs are supposed to be aired before use, how they were supposed to hang the nets and tuck in for daily use, and maintain them by washing periodically and mending torn nets. Some comments from caregivers of children under five years regarding self-efficacy are shared below:

*“... they told us that, when you want to fix it, do not fix it in the room immediately upon removing it from the rubber. But instead, you’d air dry it under a shade for 24 hours before you can remove it and fix inside. But when you fix it in the room immediately, it will make your skin hot [uncomfortable] or itchy and it will bring you a disease. So, they explained it to us as such” (R9, Caregivers FGD* *,* *Kpetoe)*

*“They said, once you are using it, you don’t have to wash it frequently. In a year, you can wash it twice. When it is dirty, we are encouraged to use key soap or Geisha”(R8, Caregivers FGD, Kpetoe)*

*“When they were coming to distribute it, they made us aware how we can take care of it. They said, when you want to wash it in a year, you wash it twice. If you want to do so too, you use key soap or Geisha to wash it and dry it under a shade.....You can sew the LLINs when it is torn so it can be in a good shape before you use it”(R9, Caregivers FGD, Kpetoe)*

*“When it is torn; we are to sew it. When we fix it too we have to tuck it properly. So, mosquitoes cannot penetrate and bite us”* (*R12, Caregivers FGD*).

#### Knowledge-practice gaps

Despite demonstration of knowledge, participants across the various communities also acknowledged inadequate behaviour change regarding LLIN use. Some community members either use the nets for fencing their farms or for drying items:

*“A lot of people collect the net and because they have gardens, they use the net to fence them instead of sleeping under the net. The government has spent money to give the nets and if you are seen using the net to fence your garden you will be sanctioned because you are misusing the net and wasting government money. The purpose for the net is for us to sleep under it to reduce malaria. There is no benefit in using the net on your garden the government should punish you if you are caught”* (R6, *Caregivers FGD, Apoli-Ningo)*

*“Some people take the net and use it for gardening and others use it to dry their palm kernel” (R1, Caregivers FGD* *,* *Achiase)*

*“…some take it and use it for different purposes which is not good. If you don’t want it don’t go for it, you can’t go for it and use it to fence a garden, it is not because of a garden they gave it to you” (R2, Household Heads FGD, Kukurantumi,)*

In some communities, however, household heads held the perception that nets were distributed during the campaign without education on its proper use and care, which could lead to misuse of the nets:

*“They didn’t give any education on the net, they just came and dump it to us and left so most of us didn’t know its use so we used it for farming and covering our things so they should have gathered” (R2, Household Heads FGD, Hokpeta-Takla).*

To address this problem, some community members proposed that the campaign messages include fear appeal content and punitive measures to deter people from misusing or requesting extra nets. For instance, they suggested assigning security agents to punish community members who collect the LLINs but misuse them:

*“ If you want us to take the campaign seriously you should employ the soldiers and the police to scare us. So if you see using the net as a fence or using it for drying purpose you will be arrested that will bring fear and even if you child is using it on the floor you will quickly snatch it from them knowing that the police can arrest you” (R5, Caregivers FGD, Achiase)*

*“You should add that everyone who receives the nets and comes back for another one will be arrested by the police because they want to steal from the government” (R1, Caregivers FGD, Apoli Ningo)*

Although community members were told that there would be ongoing monitoring for net misuse, they mentioned that this did not happen in the past:

*“It was said that if you are caught using the net for fencing you will be arrested by the police but no one come around to check if the right thing is done or not. So this year if possible promise they should try and fulfil it. Some people have net that are not spoiled so they come for the new ones to fence their gardens because they cannot buy a net. The should not notify us that they are coming around, because if I am told they will be checking the nets and I have some fencing my garden I will remove it so they come they will not see anything. They should come unannounced to check and that will put fear in the people and they will take better care of the nets” (R1, Caregivers FGD, Apoli-Ningo)*

### Pedagogy domain

The pedagogy domain includes how interventions are delivered through outreach, modelling, demonstrations, personal risk, and teachable moments [14]. In this regard, community members mentioned various ways through which campaign messages were delivered to them. These included outreach (i.e., house-to-house interactions), illustrations, and demonstrations. For instance, participants mentioned that during the distribution, the campaign implementers demonstrated how the nets are supposed to be hang. When individuals did not know how to hang them, the campaign team followed-up to their homes:

*“They came to illustrate how we were going to fix it under the tree [outreach point].” And we witnessed it. And we knew that was how we were going to fix it. So, they taught us before they now came to share it*.” *(R1, Caregivers FGD* *,* *Kpetoe)*

*“A* *t my place after receiving the net they ask you if you know how to use it, if you don* *’* *t, they will come and do it for you* (*R3,* *Caregivers FGD Hokpeta* *-Gbogame* *)*

Community members were further educated on malaria prevention and the appropriate use of LLINs through house-to-house outreach sessions. Specific outreach sessions for the elderly were also encouraged in the communities:

*“They came to teach us. For instance, on malaria, we should not dirty up our households; behind our houses, we should weed them. So that, there will not be any weed that will breed mosquitoes and be a nuisance to our children. If there is water in any cans around, mosquitoes can breed in them. And this brings malaria to our children. So, we should see to all those things; that’s what they talk on” (R8, Caregivers FGD, Hokpeta-Takla)*

*“Sometimes they come house to house to educate us on how to prevent malaria for ourselves and children. If you have empty tins of milk or tin tomatoes you have to tie them in rubber and dispose them at the refuse dump but if you leave them around and it rains in them they breed mosquitoes and the bite you. Also they tell us to sleep under the net, and teach us how to enter the nets at night and how to tuck them in the bed so mosquitoes do not enter”(R1, Caregivers FGD, Apoli Ningo)*

*“Most elderly people do not know how to fix the net so like my sister said there should be a demonstration done to teach us how to fix the different types of net. I think it will help”(R3, Caregivers FGD, Achiase*)

Caregivers described several teachable moments they experienced during the campaign. Some household heads were concerned that the education sessions focus more on women and ignored men in the community:

*“They always come to educate and speak to the women of which I think is not fair, so they should come and educate the men also on some health issues…..no, they just distributed the nets to us and asked us to use it ...”( R4, Household Heads FGD, Tsito)*

#### Communicating through multiple channels

The campaign used a mix of communication channels to transmit messages. Community members confirmed receiving messages through mass media (e.g., radio/FM stations, Television) gong-gong (drum) beating, community information centres (i.e. central points established to provide information to community members through announcements) community gatherings and health facility:

*"They gave an announcement that the nets are in so maybe if they will be distributing it today, they give the announcement the previous day that the nets are in for distribution so we should all stay back home and come for it” (R1, Caregivers FGD, Hokpeta-Takla)*

*“They usually say it in the mosque that the nets will be coming, we also hear from the information centre and individually they come to the homes when they are writing the names”(R3, Household Heads FGD, Kukurantumi).*

*“When we go for weighing, they talk about it and the come to the churches to talk to about it. We also heard from the information center, they informed us when the nets will be distributed” (R9, Household Heads FGD, Kukurantumi)*

#### Strengthening campaign through continuous engagement

To strengthen the engagement, participants recommended that future campaigns should adopt more of the house-to-house pedagogy approach and extension its duration:

*“I will suggest house to house campaign which I think is the best. Some people do not have radio or television so they will not be aware of the campaign talks done there. The home visitation can be done for two weeks so that if during the first visit the people are not home they will meet them on the second visit. I think the house to house is the best compared to the others” (R2, Caregivers FGD, Achiase)*

*“I think there should be a better awareness of the campaign. The need to extend the period for the publicity for about one month. Also on the day for the distribution the announcement should be made”(R1, Caregivers FGD, Achiase)*

They also recommended diverse approaches for continuous engagement to reinforce campaign messages, such as use of local committees to continuing education the community members, the design of a manual for use by community members, and the adoption of a train-the-trainer mechanisms.

*“We have a committee in the town you can call on them to discuss issues or the nurse can work with the committee… The committee can discuss the misuse of the net and speak to the community members to change their attitude” (R9, Caregivers FGD, Apoli-Ningo)*

*“People sometimes forget the things they hear so in any gathering like in the various churches the health talks can be announced and also when they come to the clinic they are informed as well. The constant reminders will help them keep it in mind” (R8, Caregivers FGD, Apoli-Ningo)*

*“If you will spend one month to share the net, you can train for two weeks because Achiase is a big town when you train me, I can also train the others in my home. I am not sure you can train me and also train my neighbor” (R5, Caregivers FGD, Achiase)*

*“My suggestion is that they should add something like a manual for the net so we can read ourselves and know of the usage and its benefits. Unlike the previous ones they came sharing without any education and its importance and there should be penalties for people who use it for framing and other purpose instead of its original purpose to help desist others from doing such” (R1, Household Heads FGD, Hokpeta-Takla).*

## Discussion

The results show that the LLIN campaign process entailed community engagement with traditional authorities, registration and distribution, and actual implementation supported by community health volunteers. Intervention delivery included outreach, illustrations, demonstrations, and utilization of multiple communication channels. The content included information on effective malaria prevention and enhancement of their self-efficacy for net use, yet gaps in net use were still noted.

According Galbrith’s taxonomy, the implementation domain of an intervention should contain practical features that can enable the intervention to be put into operation. This include ensuring necessary support from stakeholders/gatekeepers, selection of providers, volunteers and key staff with desired characteristics, having a clearly defined target population for whom intervention is appropriate, Intervention location appropriate for target population etc.[14]. As evident in other studies, for desired behavioural changes to occur, implementers often partner with community stakeholders through the use of different methods of engagement to ensure meaningful exchange of social support [17]. Engaging communities to implement evidence-based interventions can facilitate collaboration with groups affiliated by geographic proximity, special interest, or similar situations to address issues affecting the group’s well-being [18]. The term community generally denotes a group of people with some kind of shared social identity, while the term engagement indicates an interactive relationship between a community and a research entity [5]. In the context of mass LLIN distribution campaigns in Ghana, the implementers engaged the community through meaningful, respectful, and fit-for-purpose involvement of community members [19]. Researchers note that community engagement could take many forms, and partners can include organized groups, agencies, institutions, or individuals. Collaborators may be engaged in health promotion, research, or policy making [18]. In this campaign, both traditional authority institutions and individuals were engaged in the implementation process.

In this regard, Adikhari and colleagues (2017) encourage the use of a stepwise approach to ensure the success of community engagement [20]. This involves sensitizing authorities, seeking consensus, collaborating with government and traditional authorities at various levels, and subsequently selecting and training volunteers for implementation. Similar processes were adopted in the LLIN distribution campaign in Ghana, as observed by this study. The exact nature of the individual steps and the order in which they are taken depends on the intervention and the local context [20]. Another key assumption is that communities are experts in their own lived experience and possess cultural competence, thus engaging them in the development of solutions can enhance the relevance of interventions and facilitate uptake and sustainability [16, 17]. Consequently, collaboration between stakeholders will need to acknowledge the knowledge and skill sets of community partners [21]. Though LLIN distribution campaign in Ghana contain practical elements necessary for an effective intervention implementation at the community-level, this study however highlights challenges within the implementation domain of LLIN distribution campaign in Ghana which include insufficient training for volunteers and community attitudes towards volunteers which can influence the desired outcome of the LLIN distribution campaign. These challenges (health worker challenges) which includes inadequate training have also been documented in other studies as barrier to LLIN use [21, 22]. Hence intervention implementors must invest in capacity building and adequate training of health workers and volunteers towards achieving desired health outcomes.

The content domain entails what is been taught by the intervention which aligns with theories and models of behaviour change. This includes influencing social norms for desired behavior, intervention content that is appropriate for target population, influencing self-efficacy of desired behavior, influencing cognitions for positive behaviors, providing skills training for correct use of risk-reduction supplies or techniques among others [14]. The content of the mass LLIN distribution campaign highlights how messages for malaria prevention were designed and promoted. This study shows that the content included information on effective malaria prevention and information which enhanced community members’ ability to practice (self-efficacy); yet there are gaps in the behavioural intention in terms of net use at the community level. Health promotion messages require the identification of best practices, which includes the acknowledgement of contextual differences [23]. Best practices show that interventions’ effectiveness is based on the extent to which complex interactions are considered for the health messages. This means allowances must be made for the uniqueness of settings across time and space [24]. This creates opportunities to situate practice in its social context, optimize interventions for specific contextual contingencies, and target crucial factors influencing behavior [23]. For instance, this study suggests the use of fear appeal and punitive measures as additional strategies that could enhance proper use of the LLINs at the community level. The bulk of health promotion practice has been oriented to communityy settings and seeks to increase the sophistication with which knowledge about settings is mobilized in the planning, implementation, and evaluation of health promotionn [23]. Investing in the development of culturally appropriate malaria prevention messages is therefore crucial in increasing community awareness on malaria prevention and behavioural intention of LLIN use [25].

The pedagogy domain also referred to as educational strategies of Galbraith's taxonomy entails how the intervention is taught or delivered, or the engagement style used to convey content of the intervention such as delivery of intervention using multiple strategies, demonstration/modeling, outreach, social marketing/mass media, e.g. TV, radio, billboards, empowering target audience has ownership of intervention etc. [14]. Though the study revealed the LLIN campaign distribution in Ghana utilize outreach, illustrations, demonstrations and communicating through multiple channels, findings from this study also suggest the need for continuous educational strategies to support the campaign beyond its implementation phase. Specifically, we propose that SBCC activities be executed before the campaign, during and immediately after the campaign in the Ghanaian context. Some of the proposed channels identified for this purpose include the posters, community information centres, home visits and in churches and mosques. This would ensure that communities are well sensitised before and after the campaign. Other community-based studies indicate that, although health facts are known by community members, the majority did not practice them, which is attributed to lack of continuous awareness creation [26]. To overcome this lack of follow-through, there is the need to have interventions that seek to deliver messages repeatedly at the community level, beyond the campaign period. For this reason, we have recommended continuous delivery of health-talks as a part of community-based interventions through voluntarily recruited community members [12]. This is also evident in other studies that indicated that, sustained education on LLIN use and the use of SBCC strategies can improve malaria prevention and treatment behaviours (LLIN use) among community members [10, 26, 27]. It is worth noting that our larger project established the Community Health Advocacy Team (CHAT) within the study sites, which was aimed at sustaining the momentum for the LLIN distribution campaign in communities. The role of the CHAT is centred on key elements of community/social mobilisation and capacity building, all nested in a social and behaviour change communication strategies [28].

The findings from this study can guide similar evidence-based interventions in resource-constraint settings to effectively implement health interventions taking into consideration lessons learnt and the required core elements needed for the successful implementation of an intervention to achieve the desired health outcome.

### Study limitations

Although this study highlights critical components of the mass LLIN distribution campaign, there are notable limitations. This is a qualitative study therefore the findings cannot be generalized to other settings. However, lessons learned may be applicable to other resource- limited settings where mass LLIN distribution campaigns are implemented. Also, data collection was conducted as a pre-point LLIN mass distribution campaign activity. This means participants’ ability to recall the events of the previous LLLIN mass distribution activity and provide accurate data may be largely limited by the lapse of time. Moreover, the data collection and analysis could not be conducted ahead of the LLIN distribution campaign so as to inform execution of the 2021 distribution campaign. In this regard, we hope that these important findings would inform the implementation of the next LLIN mass distribution campaign to be executed in Ghana.

## Conclusion

Under the implementation domain, campaign participants recounted the registration and distribution processes, preceded by engagement with traditional authorities for community entry processes, and continuous involvement of community health volunteers during implementation. For pedagogy (educational strategies), the intervention was delivered through outreach, illustrations, demonstrations, and the use of multiple communication channels. Although, the content included information on effective malaria prevention, whist enhancing their self-efficacy, participants noted gaps (e.g., misuse) in the desired behavioural intentions regarding LLIN use.

Although the implementation of the LLIN distribution campaigns exhibits components for core elements of evidence informed interventions, there are gaps in the behavioural change intentions. Future campaigns in Ghana may consider use of continuous innovative pedagogical approaches at the community level to reinforce appropriate use of nets that are distributed.

### Supplementary Information

Supplementary Material 1.

## Data Availability

No datasets were generated or analysed during the current study.

## Abbreviations

CHAT: Community Health Advocacy Team
CG: Caregivers of Children Under 5 years
EPI: Expanded Immunization Programme
GHS: Ghana Health Service
HH: Household Heads
LLIN: Long lasting insecticide treated nets
NMCP: National Malaria Elimination Programme
NMEP: National Malaria Elimination Programme
PMD: Point Mass Distribution
SBCC: Social Behaviour Change Communication
