# Supplementary material for: Assessing LLIN distribution implementation using evidence-informed intervention core elements: a qualitative study in a resource-constrained setting
Source: BMC Health Serv Res. 2024 Jul 9;24:790. doi: 10.1186/s12913-024-11223-5 (PMC11234562; doi:10.1186/s12913-024-11223-5)
Supplement: Supplementary file 1 — Supplementary Material 1. [file 12913_2024_11223_MOESM1_ESM.docx]

**Qualitative study to explore the barriers and facilitators to insecticide treated net (ITN) use in Ghana**

**Focus Group Discussion Guide**

**Introduction:**

Welcome. Thank you for taking the time to join in our discussion about malaria prevention in Ghana. My name is (insert name here) and I am part of the research team for this study. We would like to learn more about net use in Ghana to help improve malaria prevention programs.

There is no right or wrong answers. We expect that you will have differing points of view. Please feel free to share your opinion even if it is different from what others have said.

We are tape recording the discussion because we do not want to miss or forget your comments. No names will be included in the final report, and your comments here will be kept confidential. Please remember that we are just as interested in negative comments as we are in positive comments, because both can be very helpful to us.

Do not feel like you are only speaking to me. Please feel free to have a conversation with each other about these questions and issues that come up. I am here to ask questions, listen and ensure that everyone has a chance to voice his or her opinion. We want to hear from each of you. If you are the only one doing the talking, I might ask you to let others speak their mind. If you are not saying anything, I might ask you to express your opinion on the subject.

Does anyone have any questions before we begin?

1. **Warm-up Question**

What are some of the key health issues in this community?

1. **Malaria-Perceived risk**

Tell me about malaria in this community.

Are there any differences in malaria during different times of the year?

What activities put people at risk of malaria?

How does malaria now compare to malaria in the past?

Who is most impacted by malaria in this community?

How easy or difficult is it for people to get treatment for malaria in this community?

1. **Context of Malaria Prevention**

How do people in this community prevent malaria?

What are some of the challenges for preventing malaria?

When did people in this community first start using insecticide treated nets?

Where have you received information about preventing malaria? How often do you hear messages about this?

1. **Motivators and Timing of Net Use**

What are some reasons people sleep under a bed net, when they have one available to them?

What months do people in your community use bed nets?

How do people decide what time of the year to use bed nets?

What part of the night do people generally use bed nets?

How do people decide what time of the night to use bed nets?

-How does this compare between men and women?

-How does this compare between children and adults?

-How about teenagers?

1. **Barriers to Net Use**

What are some reasons people do not sleep under a bed net when they have one available to them?

- Are there any differences between men and women?
- How about between adults and children?

What are some the times when people do not use bed nets?

- What makes ITN use challenging in those situations?
- How does this compare for men, women and children?
- Are there any times where it is not worth it to use a net?

What reasons might cause people to spend the night away from home?

- - Which members of the family are more likely to sleep away from home?
  - Tell me about net use when away from home
  - Is there anything that is used besides bed nets to prevent mosquito bites/malaria -away from home?

How often do people in your household sleep outside of their rooms or huts?

- - Which members are most likely to sleep outside of their rooms or huts?
  - What time of year are they most likely to sleep outside of their rooms or huts?
  - How much of the night do they spend outside of their rooms or huts?
  - What are the reasons people would sleep outside of their rooms or huts?
  - How are mosquito bites prevented outdoors of their rooms or huts?
  - Tell me about net use when sleeping outdoors of their rooms or huts
  - Is there anything that is used besides bed nets of their rooms or huts?

1. **Overcoming Known Barriers to Net Use**

- What more can be done to prevent malaria?
- For those who sleep under an ITN, how did you develop the habit?
- Tell me about someone who did not used to use a net, but started using one.
- What do you have to say about ITN use among people living in urban and rural areas?
- When is it impossible to use a net?
- What would need to change for every person to sleep under a net every night throughout the year?
- For people who are not sleeping under nets, what can be done to motivate them to use a net?
  - You mentioned X barrier (refer back to barriers mentioned earlier in discussion, e.g. heat), how do some people use nets in spite of X?
  - What can be done to address X?
  - What can we do to make it easier for people to sleep under their net?
  - What can be done to make ITNs more comfortable to sleep under?
  - Are there any differences for men and women?
  - How about for children compared to adults?
  - How about teenagers?

1. **Supplemental Questions (Only for community leaders and health workers)**

How has ITN use been promoted in your community?

- - Tell me about some of the things that worked well.
  - What were some of the challenges?
  - What are some reasons ITN promotion has been successful/unsuccessful?

What additional measures would you recommend for improving malaria prevention in Ghana?

What additional measures would you recommend for increasing net use in Ghana?

1. **Free Listing and Ranking (Guide C)**

(**Those who cannot write will be assisted by research assistants**)

8a. Could you list all the factors that make people sleep under bed net in this community?

8b. Could you rank the factors you have listed, starting from the most important to the least important?

8c. Could you list all the factors that make people not to sleep under bed net in this community?

8d. Could you rank the factors you have listed, starting from the most important to the least important?

**Wrap-up**

Is there anything we have not yet discussed today that you would like to add?

Is there anything you would like to ask me?

**Closing:** Thank participants for their time and contributions to the study
